# English Suicide-Fields, and the Restraint of Suicide

**Published:** 1862-10

**Authors:** J. N. Radcliffe


### Art. VIII.—ENGLISH SUICIDE-FIELDS, AND THE RESTRAINT OF SUICIDE.

By J. N. Radcliffe.

In a paper contributed to the previous series of this Journal,* I pointed out several curious and not uninstructive facts connected with the comparative prevalence of suicide in the different counties of England and Wales. The data from which these facts were derived extended over the three years 1856-57, and -58, and were obtained from the Judicial Statistics published by the Home Office. The fact of greatest interest elicited was, that there were three districts in which the amount of suicide was in excess of the general average of the kingdom. I proposed, for the purpose of more readily fixing this fact in the mind, to designate these districts *Suicide-Fields*. An analysis made by Dr. Farr, twenty years ago, of the proportion of suicides in the different Registration Districts, shows that the localities of greatest prevalence of the crime in the two years 1838-39 were the same as in 1856-58. It may be inferred, therefore, that the causes leading to this peculiarity of distribution are of no transitory character.

At the late Congress of the National Association for the Promotion of Social Science, I brought this subject before the Crime and Reformation Section. Since the publication of the paper to which I have referred, the official returns of suicide for 1859 and 1860 had been published, and I was thus enabled to make use of more extended data than those from which my previous deductions had been derived. In all essential respects, however, the results were similar. The counties in which the proportion of suicide was in excess of the general average of the kingdom, and the districts of inordinate prevalence of the crime, were the same. Such differences as existed, I purpose here briefly to set forth; adding some observations on the restraint of self-murder.

In the previous analysis, Westmoreland showed the greatest excess of suicides, Middlesex standing second in order of amount; while the least proportion was observed, among English counties, in Cornwall and Cambridge; among Welsh, in Pembroke.

In the more extended analysis, Middlesex stands at the top of the suicide scale; Westmoreland holds the second place in point of excess; while, at the bottom of the scale, among Eng-

* On the Distribution of Suicides in England and Wales.—*Journal of Psychological Medicine*, vol. xii. p. 469.

*TABLE showing the Distribution of Suicides in the different Counties of England and Wales, during the Five Years 1856—60.*

| COUNTIES. | 1856. | | 1857. | | 1858. | | 1859. | | 1860. | | Annual Average of Suicides. | Estimated Average Population. | Average of Suicides to 100,000 Population. | Annual Average of Persons to 10,000 Population who signed the Marriage Register with marks, 1856—1858. |
|---|---|---|---|---|---|---|---|---|---|---|---|---|---|---|
| | Males. | Females. | Males. | Females. | Males. | Females. | Males. | Females. | Males. | Females. | | | | |
| Bedford | 4 | 7 | 2 | 4 | 2 | 2 | 5 | 1 | 8 | 6 | 8·2 | 132,024 | 6·2 | 70·4 |
| Berks | 6 | 8 | 1 | 2 | 9 | 3 | 5 | 2 | 8 | 6 | 10·0 | 174,280 | 5·7 | 52·4 |
| Buckingham | 11 | 5 | 3 | 2 | 8 | 1 | 11 | 1 | 4 | 0 | 9·2 | 165,732 | 5·5 | 45·3 |
| Cambridge | 5 | 4 | 2 | 1 | 1 | 2 | 5 | 1 | 6 | 1 | 5·6 | 178,790 | 3·1 | 56·7 |
| Cheshire | 21 | 39 | 3 | 15 | 21 | 9 | 23 | 6 | 30 | 10 | 35·4 | 490,319 | 7·2 | 56·9 |
| Cornwall | 8 | 5 | 2 | 3 | 7 | 5 | 11 | 2 | 3 | 4 | 10·0 | 365,190 | 2·7 | 61·2 |
| Cumberland | 14 | 14 | 4 | 3 | 13 | 4 | 11 | 4 | 14 | 7 | 17·5 | 202,352 | 8·6 | 30·7 |
| Derby | 14 | 24 | 8 | 8 | 13 | 5 | 16 | 3 | 18 | 9 | 23·6 | 326,387 | 7·2 | 43·0 |
| Devon | 31 | 10 | 11 | 13 | 28 | 10 | 31 | 11 | 34 | 8 | 37·5 | 579,299 | 6·4 | 42·7 |
| Dorset | 8 | 2 | 2 | 5 | 5 | 4 | 6 | 4 | 4 | 2 | 8·5 | 187,316 | 4·5 | 45·7 |
| Durham | 16 | 24 | 9 | 7 | 15 | 10 | 12 | 9 | 21 | 5 | 25·6 | 473,611 | 5·4 | 71·8 |
| Essex | 12 | 16 | 5 | 2 | 19 | 11 | 11 | 3 | 13 | 5 | 19·5 | 394,056 | 4·9 | 44·3 |
| Gloucester | 23 | 21 | 6 | 13 | 23 | 7 | 29 | 5 | 19 | 8 | 30·8 | 477,488 | 6·4 | 45·0 |
| Hants | 26 | 34 | 6 | 6 | 22 | 7 | 22 | 10 | 22 | 4 | 31·8 | 458,654 | 6·9 | 40·6 |
| Hereford | 5 | 7 | 3 | 1 | 0 | 1 | 1 | 2 | 2 | 0 | 4·4 | 121,208 | 3·6 | 42·5 |
| Hertford | 4 | 11 | 4 | 2 | 4 | 1 | 9 | 4 | 4 | 2 | 9·0 | 171,491 | 5·2 | 52·7 |
| Huntingdon | 1 | 4 | 1 | 3 | 0 | 0 | 1 | 0 | 0 | 0 | 2·0 | 64,260 | 3·1 | 51·6 |
| Kent | 51 | 41 | 19 | 16 | 52 | 16 | 52 | 16 | 51 | 27 | 68·2 | 698,296 | 9·7 | 37·9 |
| Lancaster | 136 | 129 | 54 | 46 | 114 | 36 | 129 | 36 | 130 | 33 | 168·6 | 2,309,486 | 7·3 | 82·0 |
| Leicester | 14 | 20 | 6 | 3 | 20 | 6 | 11 | 2 | 20 | 3 | 21·0 | 235,878 | 8·9 | 48·1 |
| Lincoln | 23 | 24 | 15 | 13 | 24 | 10 | 23 | 10 | 26 | 12 | 36·0 | 410,561 | 18·7 | 41·7 |
| Middlesex | 144 | 159 | 69 | 89 | 140 | 58 | 165 | 58 | 148 | 84 | 24·8 | 2,110,009 | 0·5 | 29·7 |
| Monmouth | 5 | 8 | 1 | 0 | 5 | 4 | 6 | 2 | 4 | 3 | 7·6 | 169,493 | 4·4 | 103·0 |
| Norfolk | 16 | 30 | 14 | 9 | 8 | 4 | 14 | 9 | 13 | 9 | 25·2 | 437,611 | 5·7 | 53·8 |
| Northampton | 12 | 8 | 3 | 5 | 9 | 2 | 4 | 2 | 8 | 4 | 11·4 | 223,118 | 5·1 | 55·5 |
| Northumberland | 10 | 14 | 9 | 8 | 11 | 8 | 4 | 1 | 9 | 4 | 15·6 | 331,190 | 4·7 | 45·6 |
| Nottingham | 16 | 18 | 5 | 9 | 26 | 7 | 20 | 7 | 14 | 3 | 25·0 | 286,772 | 8·7 | 71·1 |
| Oxford | 5 | 7 | 2 | 2 | 7 | 4 | 7 | 4 | 5 | 0 | 8·6 | 171,713 | 5·1 | 47·0 |
| Rutland | 1 | 1 | 2 | 0 | 0 | 0 | 0 | 0 | 0 | 0 | 0·8 | 22,199 | 0·3 | 27·0 |
| Salop | 13 | 5 | 1 | 2 | 11 | 3 | 8 | 1 | 12 | 6 | 12·4 | 237,412 | 5·1 | 63·9 |
| Somerset | 18 | 11 | 4 | 5 | 12 | 4 | 14 | 4 | 17 | 4 | 18·6 | 444,476 | 4·1 | 48·3 |
| Stafford | 26 | 14 | 5 | 4 | 25 | 11 | 14 | 5 | 21 | 11 | 27·2 | 705,218 | 3·8 | 94·2 |
| Suffolk | 16 | 18 | 3 | 2 | 8 | 6 | 20 | 13 | 23 | 5 | 22·8 | 336,543 | 6·7 | 58·0 |
| Surrey | 61 | 49 | 38 | 21 | 44 | 19 | 45 | 20 | 57 | 21 | 75·0 | 786,402 | 9·5 | 30·7 |
| Sussex | 20 | 23 | 8 | 9 | 31 | 7 | 17 | 9 | 29 | 7 | 32·0 | 355,604 | 8·9 | 30·8 |
| Warwick | 28 | 34 | 14 | 14 | 28 | 16 | 24 | 5 | 33 | 19 | 43·4 | 535,710 | 8.0 | 58·0 |
| Westmoreland | 6 | 3 | 3 | 2 | 5 | 1 | 5 | 1 | 3 | 1 | 6·0 | 60,051 | 9·9 | 22·5 |
| Wilts | 8 | 6 | 4 | 3 | 9 | 3 | 13 | 6 | 7 | 3 | 12·4 | 250,882 | 4·9 | 44·3 |
| Worcester | 10 | 12 | 3 | 1 | 7 | 5 | 12 | 6 | 19 | 2 | 14·8 | 298,395 | 4·9 | 60·2 |
| York | 73 | 71 | 30 | 25 | 94 | 42 | 72 | 34 | 84 | 40 | 113·0 | 1,815,530 | 6·2 | 59·2 |
| Anglesey | 0 | 1 | 0 | 1 | 1 | 3 | 1 | 0 | 0 | 1 | 1·6 | 55,381 | 2·8 | |
| Brecon | 3 | 5 | 1 | 0 | 4 | 1 | 2 | 0 | 4 | 3 | 4·6 | 61,579 | 7·4 | |
| Cardigan | 1 | 1 | 4 | 1 | 1 | 0 | 0 | 2 | 1 | 1 | 2·4 | 71,811 | 3·3 | |
| Carmarthen | 3 | 2 | 0 | 1 | 0 | 0 | 1 | 1 | 2 | 2 | 2·4 | 111,416 | 2·1 | |
| Carnarvon | 3 | 4 | 1 | 1 | 2 | 0 | 0 | 3 | 1 | 1 | 3·2 | 93,323 | 3·2 | |
| Denbigh | 0 | 3 | 1 | 0 | 2 | 0 | 0 | 1 | 3 | 2 | 2·4 | 98,372 | 2·4 | 78·1 |
| Flint | 0 | 2 | 0 | 0 | 3 | 1 | 1 | 0 | 1 | 0 | 1·6 | 49,353 | 2·3 | |
| Glamorgan | 3 | 9 | 3 | 5 | 11 | 5 | 6 | 9 | 9 | 6 | 11·8 | 291,979 | 4·0 | |
| Merioneth | 1 | 1 | 0 | 1 | 0 | 0 | 0 | 0 | 0 | 0 | 0·6 | 38,871 | 0·1 | |
| Montgomery | 3 | 2 | 0 | 0 | 2 | 1 | 3 | 0 | 1 | 0 | 2·4 | 97,143 | 3·5 | |
| Pembroke | 1 | 0 | 0 | 1 | 1 | 0 | 2 | 2 | 3 | 2 | 2·4 | 95,505 | 2·5 | |
| Radnor | 0 | 0 | 0 | 0 | 2 | 1 | 0 | 0 | 0 | 1 | 0·8 | 25,192 | 0·3 | |
| | | | | | | | | | | | South Wales ... | | 3·8 | 88·2 |
| | | | | | | | | | | | North Wales ... | | 2·7 | 62·6 |
| TOTALS | 919 | 395 | 960 | 389 | 909 | 366 | 910 | 330 | 961 | 396 | 1313·0 | 19,572,869 | 6·7 | 55·2 |

lish counties, are Rutland and Cornwall; among Welsh, Radnor and Merioneth.

The counties and districts of inordinate tendency to suicide are as follows:—

| | | Proportion of Suicides to 100,000 Population. |
|---|---|---|
| *The London Suicide-Field.* | Middlesex | 10·5 |
| | Kent | 9·7 |
| | Surrey | 9·5 |
| | Sussex | 8·9 |
| | Hants | 6·9 |
| *The Midland Suicide-Field.* | Leicester | 8·9 |
| | Lincoln | 8·7 |
| | Nottingham | 8·7 |
| | Warwick | 8·0 |
| | Derby | 7·7 |
| *The Northern Suicide-Field.* | Westmoreland | 9·9 |
| | Cumberland | 8·6 |
| | Lancaster | 7·0 |
| | Chester | 7·2 |
| Brecon | | 7·4 |
| England and Wales | | 6·7 |

The accompanying Table gives the more extended and accurate data derived from the Home Office returns for the five years 1856-60. I can add nothing to what I have already said in my previous paper of the probable causes determining the peculiarities observed in the distribution of suicides in the kingdom. A wider examination of the relationship existing between the prevalence of instruction in a district and the proportion of suicides, still shows that there is a general, although not constant connexion between the one and the other; and that, as a rule, the *average number of suicides decreases as the average amount of ignorance increases.*

In France the relation of suicide to ignorance is more manifest than in this country; self-murder not only being most prevalent in the more highly educated districts, but also having progressively increased in frequency in proportion as instruction has become more diffused in each region as well as throughout the whole of France.*

It is highly probable that this relationship between the distribution of suicide and the degree of instruction in a district is not apparent merely. If the connexion should suggest a doubt as

* Lisle, *Du Suicide*, p. 81.

to the character of our boasted civilization, the fact may be pointed out, at the outset, that suicide is more frequent among several classes of artisans, than it is among better educated people. Hence, "if the progress of civilization," as Dr. Farr remarks, "is to be charged with the increase of suicide, we must, therefore, understand by it the increase of tailors, shoemakers, the small traders, the mechanical occupations, and the incidental evils to which they are exposed, rather than the advancement of truth, science, literature, and the fine arts."*

Disease, suffering, and misery are the chief factors in the causation of suicide. Insanity was the assigned cause in somewhat more than one-sixth of 4077 cases examined by Brierre de Boismont; certain other diseases were also answerable for about one-sixth; drunkenness for the same proportion; and domestic troubles, chagrin, and wretchedness, for more than one-fourth. The remaining instances, also about a fourth, were ascribed to mental emotions of various kinds.†

It is manifest that self-murder is not either a necessary or a very common result of these causes. They are, indeed, spoken of as *determining*, as contradistinguished from the influence exerted upon the production of suicide by sex, age, civil condition, occupation, poverty or wealth, morality, state of instruction, hereditary transmission, meteorological changes and climate, which are characterized as *predisposing* causes. It can hardly be doubted that the influence of these predisposing causes, with the exception of hereditary transmission, is, when not manifestly psychical, dependent upon the moral rather than the physical conditions of the different circumstances of life which the terms designating them represent. Of the hereditary transmission of a proclivity to suicide, it may be said that it is of rare occcurrence, and even then it is questionable how far the suicidal propensity is not associated with some other form of insanity, which is, in reality, the transmitted evil. Young put the question of the influence of climate upon its right foundation when he wrote,

"Immoral climes kind Nature never made."

Suicide is most common in spring and summer; but it does not follow that this arises solely, or even largely, from the meteorological character of the two seasons. Of the remaining predisposing causes, the state of instruction is the most important, for, to a greater or less extent, it modifies or governs the influence of the rest.

The researches of Brierre de Boismont show that the

---

* The *Third Annual Report of the Registrar-General*, p. 185.

† Brierre de Boismont, *Du Suicide*, p. 100.

great majority of suicides have, like criminals, a very imperfect education.* This fact probably affords a legitimate explanation of the connexion between the prevalence of suicide and education; and, further, it indicates that much of the instruction in vogue is of a very doubtful character, or is exceedingly deficient in the elements of a sound moral training.

In somewhat more than 40 per cent. (42·7) of the cases examined by M. Brierre de Boismont the morality was bad, the individuals having been drunkards, gamblers, or thieves, or lived in concubinage or adultery. "But," observes M. de Boismont, "the proportion of those individuals leading a regular life, and who have attempted to shorten their days, diminishes singularly, if it be recalled to mind that there are among them a considerable number of lunatics, weak-minded, and exalted spirits, hypochondriacs, sick, and persons reduced to poverty and misery. It is true, then (he adds), that the vices and passions exercise an unfavourable influence upon our acts and determinations, and that those who abandon themselves to them have in prospect poverty, a prison, sickness, insanity, and suicide."†

Here, then, in the conjunction of defective or perverted morality with imperfect instruction, we obtain a clue to the chief differential element in the causation of suicide.

It is not difficult, perhaps, to conceive in what manner a lack of morality or of moral training and imperfect instruction foster suicide; for whatever tends to weaken our notions of the sinfulness or criminality of the deed will of necessity render man most prone to it. "The suicide," Aristotle wrote, "does not undergo death because it is honourable, but in order to avoid evil;"‡ and Plato has expressed a like sentiment.§ The suicide, indeed, believes death to be an evil of less magnitude than the ill from which he seeks to escape. This is the great principle which governs the commission of self-murder. No influence, therefore, (as I have elsewhere said,||) can be conceived more powerful to hold in check the self-infliction of death than such as indelibly fixes upon the act the character of an evil far surpassing in gravity any, even the sharpest or bitterest, ill to which man may be subjected. Hence the Christian Church, in branding suicide as an inexpiable offence against God, and Christian legislatures, in attaching to it the stigma of an irreparable and disgraceful crime towards man, stamp the act as an evil, morally and socially,

* Of 3,086 cases of suicide, 25·5 per cent. read and wrote well, 53·7 per cent. read and wrote without orthography, or read without writing, while in 18·5 per cent. only was the education good, and 2·1 per cent. were entirely without instruction.—*Du Suicide*, p. 85.

† *Du Suicide*, p. 87. ‡ *Ethics*, book iii. c. 7. § *Phædo*.

|| The Æsthetics of Suicide.—*Medical Critic*, vol. i. p. 486.

to which all others which might prove influential in preventing self-murder are, in comparison, petty and insignificant.

That an imperfectly developed or depraved morality should enfeeble our notions of the sinfulness and criminality of suicide will be conceded; and a little reflection will show that these notions are much more likely to suffer when an individual has been subjected alike to defective instruction and defective moral training. For, to look at the question from one, and most restricted point of view only, when the education has advanced so far as to give birth to a taste, however imperfect, for the works of our standard, or more meritorious authors, ancient or modern, the nobility of thought which so commonly pervades their writings supplies to some extent the teachings of a more systematic moral schooling. But when an education has been so imperfect as to consist of the merest rudimentary instruction, the mind being at the same time insufficiently ballasted by moral truths, this too frequently happens :—the ability to read, in which that instruction chiefly consists, serves as a pander to the passions, the mind being fed mainly upon the most trashy romance literature of the day, or seeking its prime enjoyment in the more exciting events detailed in the current news. Persons thus tutored gloat upon the horrible records of murder, suicide, and brutality, to which the newspaper press gives unsparing publicity, and constitute the richest soil for the fruition of specious theories of politics and social life. Belonging to sections of the population which, more than any other, are subject to the sufferings arising from mutations of trade, and to the hardships and discomforts of life, the susceptibilities of such individuals to the ills that may befal them are too often exaggerated by the false estimate of life, and of the duties of life, to which their mental indulgences conduct them; and this increased susceptibility renders them less able to contend against the temptations to wrongdoing which may assail them. They also, in common with the most ignorant, are principally held in check by the conventional usages of society, and the dread of the law ; and, in respect of suicide, by the fear of death as a physical evil. But these motives exercise their influence more after the fashion of traditional superstitions than as rational rules of conduct; and, powerful though they may be, they form but imperfect barriers against the impulse of vehement passion, and still less formidable obstacles against the slow but surer sap of protracted ills. Yet their controlling power is greater in the entirely ignorant than in the imperfectly educated man, from the defective instruction of the latter, when not duly curbed by more careful moral training, becoming a peculiar and special additional means of mental and moral vitiation, as the criminal records of every civilized country most surely show.

In the production of no single crime is the baneful influence of an imperfect education more apparent than in suicide; since it not only predisposes to the act by weakening generally the great moral restraints which fence it in, but in a more marked manner by giving birth to a special proclivity to it.

It would be difficult perhaps to overstate the feelings of sympathy and pity which are excited in the minds of large masses of the population by the detailed recitals of suicides, which are so constantly met with in the pages of the newspaper press, and which form a stock feature of much of the romance literature in vogue. The circumstances which usually attend and lead to the suicides of every-day life—disease, suffering, and misery—are, alas! of such a common-place and familiar character, that they appeal most powerfully to the sympathies of many who know them from bitter experience. The fact of suicide is again and again brought before the mind, and the notion, never under such circumstances far absent, is ever apt, uncalled for, to start up under stress of ills, like the "wicked whisper" which assailed Bunyan in his passage through the Valley of the Shadow of Death. This, it will readily be believed, is much more liable to occur with those who, as the ill-instructed, seek their chief mental indulgence in recitals of the tragic events of ordinary life or in literature which is addressed to the passions; and when the insidious notion, suggestion like, concurs with suffering (it matters not, so far as the individual is concerned, whether this be mental or bodily, real or imaginary, self-induced or not), and with absence of true moral control, we need not wonder that often neither conventional usage nor the restraint of the law, nor the physical fear of death proves of any avail to check the impulse of the moment, or as most commonly happens, to withstand the wearying, exhausting, and, under such circumstances, brutalizing effects of protracted ills.

The influence of a deteriorated or depraved morality in fostering suicide is, perhaps, most clearly manifested in the history of the crime in Europe since the period of *Werther*. In studying that history, moreover, we best discover in what manner these causes affect the more highly educated classes of society. This subject has, however, been so recently discussed in the pages of this Journal,* that this brief reference to it here is alone needed.

If what I have thus far suggested be correct, it would follow that the chief means at our command for controlling suicide is

---

* See the articles on the *Classic Land of Suicide* and the *Æsthetics of Suicide*, vol. i. p. 182, and p. 563.

the maintenance in all its integrity of the opinion that the act is sinful and criminal in the highest degree. So far as the notion of the sinfulness of suicide is concerned, it may be assumed that this will depend upon the state of religious instruction in a community; and we have already seen that suicide is one of several indications that there is a serious deficiency in much of the instruction, particularly of the common instruction, existing in this kingdom. So far as the notion of criminality is concerned, it is manifest that the perfectness of this will depend upon the proper carrying out of the intentions of the law. Whilst the law required that the body of the suicide should be treated with revolting barbarity, and that his goods should be confiscated to the crown, doubtless evils arose from its administration, which, to a great extent, neutralized its influence upon the evil sought to be checked. But of late years the administration of the law has run from the extreme of barbarism to the extreme of sympathy. It is scarcely too much to assert that it is now the rule with juries, upon little more than the fact of suicide, to return a verdict of "temporary insanity." The mischief of this course is great; for at the same time that it removes the stain of criminality from the act, it also sets aside the notion of sinfulness, thus sweeping away the two great obstacles to the commission of self-murder. It begets, moreover, a seemingly legitimate ground of sympathy with suicide, which is most objectionable and injurious.

At the present moment, in fact, neither the intentions of the Legislature nor of the Church are faithfully carried into effect. This also has been the case for some time back; hence those writers who, from the great prevalence of suicide of late years, not only in this but in other civilized countries, maintain that all legislation, whether civil or ecclesiastical, is of no avail for the prevention of suicide, argue from insufficient premises.

In so far as suicide is brought about by insanity and suffering, its diminution must depend upon the diminution in intensity or prevalence of those causes. Let it not be supposed, however, that insanity is a cause of suicide *per se*. The lunatic who commits suicide seeks death, with few exceptions, in the same manner and for the same reasons as the sane individual. He also is wishful to escape from some weighty ill which besets him and bears him down, but in his case the ill is solely a delusion of, or it is unduly exaggerated by a diseased mind. Unhappily the mental disorder heightens the predisposition to self-murder, intensifies and renders less controllable the mental emotions which urge a man to the act, and is not to be relieved by moral means alone. But it is important to understand that suicide is not a necessary but an incidental consequence of insanity, and that the development of the idea of self-murder is, as a rule, dependent upon a similar series of causes in the insane as the sane. Hence, whatever tends to control the proclivity to suicide among the sane, will also, to some extent, tend to control the proclivity among the insane.

For the rest, notwithstanding the complexity of the causes leading to suicide, it is more than probable that if, in the first place, the law were fully carried out, the tendency to the act would be placed under more effectual restraint. The law brands suicide, except when connected with lunacy, as a crime, and this not to punish the self-murderer (as is sometimes absurdly assumed) but to deter others from committing the deed. So long, therefore, as coroners' juries persist in passing verdicts of "temporary insanity" upon the slightest evidence, or in the absence of all evidence, in cases of suicide, so long must the influence of the legal restraint upon the living be greatly weakened, if not entirely destroyed. But the evil of the verdict of "temporary insanity," when given upon insufficient grounds, does not rest here, for it disarms not only the civil but the canon law. The latter by refusing to the suicide the rights of Christian burial, and of interment within consecrated ground, stamps the act of self-murder with an abominable and disgraceful, as well as sinful character, even more powerfully than the civil law. The important moral influence arising from this source is, however, entirely done away with by a verdict of "temporary insanity," under cover of which the suicide receives the rites of Christian burial. Notwithstanding, therefore, the amiability of feeling which frequently prompts jurors, out of sympathy towards the unhappy suicide or his family, to avoid a verdict of *felo-de-se*, this kindliness of disposition, by weakening the two great social checks to the crime, undoubtedly fosters the commission of self-murder.

But even if both the civil and canonical law were fully carried into effect, it is not to be forgotten that their influence would be limited by the degree of moral and religious instruction existing among the people. Upon the perfecting of this instruction must of necessity depend the ultimate success of any legislative measures for the suppression of suicide, or of any other crime; these measures being but rough methods for securing that self-control which each individual should himself possess. In the absence or deficiency of this instruction, there is, however, much that may be done, in addition to what the law and the Church prescribe, to obtain and maintain a healthier tone of feeling concerning suicide. The press might with advantage restrict within straiter bounds the details it so frequently makes public of singular suicides, without sacrificing the advantages derived from a free publication of events. But this is perhaps even of less importance than divesting such details of, or guarding them from, all maudlin sentiment—of all sentiment which is not of a directly practical and sterling character. Although not underrating the dangerous influence exercised by the public relation of suicides upon perverted or morbid minds, the power of the press as a teacher is so great, and its influence for good is so incalculably important, that it is not without diffidence I suggest any restriction upon its publicity, except when such publicity would be directly offensive to morals. Still, when the details of an event simply appeal to a vitiated taste for the horrible (which, alas! we all possess more or less), and when, as in the case of suicide, such details are apt to induce in certain minds a disposition to commit the act described, we are justified in asking the press to exercise its privileges within very narrow limits. But above all we should ask the press to declare a war to the knife against those perverted sentiments which too frequently are sought to be affixed to the subject of suicide,* and which among our neighbours across the Straits find expression in a peculiar form of romance literature,—sentiments inconsistent with sound moral feeling, and which have a most poisonous, yet, withal, insidious effect upon the minds of the young and thoughtless.

If sentiment, other than what arises from a sound philanthropy, is to be imported into the subject, let it at most be after the fashion of that which the forsaken and unhappy Sparabella set forth in her woful plaint:—

" Farewell, ye woods, ye meads, ye streams that flow;
A sudden death shall rid me of my woe.
This penknife keen my windpipe shall divide.
What! shall I fall as squeaking pigs have dy'd?
No. To some tree this carcase I'll suspend.
But worrying curs find such untimely end!
I'll speed me to the pond, where the high stool
On the long planks hangs o'er the muddy pool,
That stool, the dread of every scolding quean;
Yet, sure a lover should not die so mean!
There plac'd aloft, I'll rave and rail by fits,
Though all the parish say I've lost my wits;
And thence, if courage holds, myself I'll throw,
And quench my passion in the lake below.
'Ye lasses, cease your burthen, cease to moan,
And by my case forwarn'd, go mind your own.'
The sun was set, the night came on apace,
And falling dews bewet around the place;
The bat takes airy rounds on leathern wings,
And the hoarse owl his woful dirges sings;
The prudent maiden deems it now too late,
And till to-morrow comes defers her fate."†

* The *Daily Telegraph* has set an admirable example to the press in this respect.

† Gay's Shepherd's Week—*Wednesday; or, the Dumps.*

